# Abnormal Delinquency*Read before the Department of Public Welfare, February 28, 1916.

**Published:** 1916-04

**Authors:** Minnie L. Maffett

**Affiliations:** Dallas, Texas


### Abnormal Delinquency.*

MINNIE L. MAFFETT, M. D., DALLAS, TEXAS.

The subject of the abnormal or defective delinquent, by common consent, includes persons who are insane, epileptic, feeble-minded, alcoholic, drug fiends, also those who possess such other physical or mental defects as may become contributory factors in delinquency.

I believe that we are not far wrong in concluding that delinquents are, in practically all cases, abnormal either physically or mentally, and that these abnormalities constitute a really serious problem in dealing with offenders of all ages and sexes. As to this defectiveness, we find Dr. Henderson, professor of sociology of Chicago University, after excluding the legally irresponsible—as the demented, insane, and feeble-minded, dismissing Lombroso's Criminal Born and leaving aside the disputed assertion that crime is a disease, directly or biologically inherited, saying that we may

*Read before the Department of Public Welfare, February 28, 1916.

be safe in making the generalization, which is confirmed by a vast number of observers in all countries, "that most criminals are physically and mentally inferior." Such inferiority, he thinks, is due to defective nutrition of nerves, brain, and muscle, thus opening up an unlimited field for the discussion of social and economic conditions generally. Another says that "underneath every crime is some kind of incompetence, and underneath incompetence is some kind of physical defect, either inherited or acquired."

Tarde, the critic of Lombroso, also says that the criminal is before everything else a sick man, "and that the final formula is insufficient nourishment of his nerve centers—badly nourished brain, misfortune, poverty." "This," he says, "is all that remains of the criminal type." I may say here that I consider poverty, filth, bad housing, bad associations, etc., of the greatest importance in the causation of crime. Still I am thoroughly convinced that these physical and mental factors are certainly of sufficient importance to justify energetic measures for their control, both for the welfare of the individual and of society at large.

Travis gives a rather interesting history of our present ideas of and interest in these physical causes in his study of juvenile delinquency in "The Young Malefactor." As you know, the Italian school claims that the criminal, and by implication, the delinquent, is a person marked by typical characteristics of body and mind. In remote times an extraordinary man was supposed to have an unusual body, and an ugly body was associated with a correspondingly bad man—so much so that mediaeval law actually punished the uglier of two suspects. Almost all founders of religion were supposed to have superhuman births. Buddah is described as having "long arms, hairy limbs, forty-two teeth, flat-footedness, long ears, and high nostrils"—probably the almond-shaped nostrils more recently observed. All of these were described by Lombroso as stigmata of crime. There are many references in Hebrew literature to unusual physical characteristics of various people of note, both good and bad.

Homer describes a certain obnoxious character by telling that he was "the ugliest man who ever came to Ileum—bandy-legged, lame on one foot, his round shoulders seemed drawn together on his chest, his head, moreover, was pointed (keel-shaped) and sparse was the wool that grew thereon." Plato declared that the wicked owe their wickedness to their physical organization—thus recognizing congenital tendencies—while Hippocrates, Aristotle, and Galen made similar observations which later became of such common knowledge that various proverbs arose, such as "from the visage, one knows the vice." Various men collected a lot of data of more or less interest and accuracy until the middle of the nineteenth century, when Darwin advanced his theory of evolution, which was accepted by Lombroso and by whom this data was woven into the more modern stigmata theory by which the Italian school claimed not only to be able to discover a criminal, but also to tell what crime he has committed, and to even forecast correctly the crime which he would commit if he had not already reached the criminal stage. Some of these abnormalities are a symmetry of the skull, projecting ears, thick head of hair, and thin beard, prominent cheek bones, frequent gesticulations, various anomalies of the palate, teeth, and a host of others. A criminal is supposed to have five of these, and to be a murderer if the fates have saddled six or seven of them on him. In investigating a large number of students and popular audiences, Travis found that when five or more of these were found in the same individual, the family history revealed insanity, epilepsy, a neurotic tendency, or special ability in some line, not necessarily morbid. A summary of the investigations of a number of the more prominent observers leads to the conclusions that these stigmata are found in greater numbers and in more suggestive combinations in older offenders, but in not more than 10 per cent of these cases do they occur in such striking combinations as to suggest natural criminality or types of crime. These stigmata were found in gradually decreasing numbers, as the children in the various graded institutions in New York State were examined, showing an encouraging absence of marked stigmata in those of the milder offenders and we seem justified in concluding that these are really more marks of denegeration than of crime alone.

Another interesting observation as to the general physical inferiority of the criminal as compared to the average American, is made by Dr. Sylvester, superintendent Wisconsin State Hospital and formerly physician in charge of Wisconsin State Prison Hospital, in a study of 1521 prisoners in that institution. Taking the average for 221,000 men examined by the forty-three old-line life insurance companies as representing the average American citizen, he found these prisoners, on an average, 1.8 inches below this standard as to height, while the recidivists studied lacked 2.5 inches. Also in this latter class is found the highest percentage of the other physical stigmata, as degenerate palates, cranial asymmetry, etc. He also finds that diseases of the chest, as T. B., pigeon breasts, stoop shoulders, etc., are the rule, heart lesions common, defective sight in 33 per cent of the prisoners; that 50 per cent admit venereal infection; 50 per cent have used alcohol, and only 10 per cent claim to have been total abstainers. It must be remembered that these conditions are probably partly the result of poverty, bad housing, and unhygienic living conditions, and as such, are as much the end-results as the causative factors in the delinquency of this group of cases. The family histories of his group are interesting and suggestive, showing:

Epilepsy, 13 per cent.
Insanity, 19 per cent.
Feeble-minded, 12.1 per cent.
Other crimes, 15.9 per cent.
Tuberculosis, 21.6 per cent.
Intemperance in father, 36.8 per cent.
Suicides, 3.7 per cent.

In a group of more confirmed criminals serving two or more years, these percentages were in most cases much higher than those given above.

In discussing the physical basis of crime from the standpoint of a judge of the juvenile court, Judge Edward F. Waite of Minneapolis says that "whenever comparison has been made, the proportion of physical defectives is found to be greater among juvenile delinquents than among school children generally. In a fair series of representative cases from his court examined, 87 per cent were in some way defective. Of 124 first offenders 84 per cent, and of 80 repeaters studied 90 per cent had some physical defect, while careful medical supervision in the Minneapolis schools gave 71 per cent as possessing some physical defect. This seems to me highly suggestive of the part played by physical deficiency in the etiology of delinquency in these cases."

Some of the more prominent defects that may be directly causative, or at least lead "toward crimes" are enlarged tonsils, and adenoids, phymosis, bad teeth, defective vision, venereal diseases, and of greater consequence and more direct bearing, epilepsy and feeble-mindedness. As to the sequence of adenoids in this connection, we may have mouth breathing, loss of hearing with accompanying loss of mental alertness and acuteness, inattention and restlessness in school and at home, irritability and dullness, rebukes and punishments, loss of grade, truancy and idleness, the gang, and finally lawlessness and crime. That such conditions are retarding factors in school, and as such, are responsible to some extent for delinquency, is shown by Dr. Josephine Baker, director of the Bureau of Child Hygiene, New York City, who found mouth breathers in only 7.6 per cent of the routine examinations and in 20.4 per cent of the retarded children in the public schools in that city. Phymosis produces local irritations, which often lead to vicious practices that in time tend to lower the general moral tone, weaken the will, and result in sexual immorality and crime. Sex delinquency in girls may have its origin in quite similar conditions.

Venereal diseases have a definite right to consideration here when we find Plant claiming that 33⅓ per cent of the children of syphilitics inherit the disease. Inherited syphilis, as you know, manifests itself in arrested development of the emotional and volitional spheres more than as an intellectual defect, both being definitely characteristic of delinquents. The late, or tertiary stage, brings general paresis, cerebral syphilis, etc., characterized by mental deterioration, depression, and delusions of various kinds which often lead to criminal acts for which they can in nowise be held morally responsible. It will be remembered that Mayor Gaynor's assailant was a general paretic.

As to drugs, their extent and train of symptoms, as lying, thieving, begging, vagrancy, prostitution, and many others, are too well known to merit much further discussion here. Defective vision, with a parallel sequence to adenoids given above, is, I believe, a factor of the greatest importance. Dr. Baker reports 9.3 per cent of such defects in the routine examinations in the New York schools, and 34 per cent in retarded children. A study of interest bearing upon the connection between retardation of school children and delinquency was made by Miss Christ of the University of Minnesota in a study of 330 delinquent boys and girls in the juvenile court of Minneapolis. In these court cases she found 70 per cent of the boys and 91 per cent of the girls retarded one year or more in school, while a corresponding retardation in the Minneapolis schools generally was only 29 per cent for the boys and 23 per cent for the girls, while Dr. Josephine Baker, in the examination of more than one-third million school children, reports 30.4 per cent as having general physical defects as compared to 78 per cent of general defects in retarded children. These results are in keeping with all others I have investigated, and, if true, argue more strongly than any impassioned appeal I could make for the early detection and correction of such defects by more careful parental and medical oversight, the passage and enforcement of compulsory education laws that work, and the formation of special classes, to be discussed later, for those who demand such care. In order that these defects may be corrected and thus remove one real etiological factor in delinquency, I cannot too strongly urge competent medical supervision in your schools —not such medical care that permits the inspector to draw a starvation salary for lining up a class of fifty to sixty children and "inspecting" them in an hour or less, but just such careful oversight as you would expect of your family physician should you consult him in a private capacity. This service must be skilled service, and as such must be paid for. A reasonable experience in institutional work has convinced me that one great reason that our Texas institutions often fall short in performing, in the largest way, the work for which they were established, lies in the inability to get and keep efficient help, and back of this the fact that the payrolls indicate a laborer's wage, while the work demands expert service. In this connection I want to enter a plea for justice and common sense in regard to the work we expect of our public school teachers. I recently noticed an indictment of teachers generally because they had, in a social service sense, failed in the recognition of "incipient" diseases in school children—a work that often taxes the most expert diagnostician and for which the teacher has no adequate means of preparation. Doctors and nurses are logically the ones to do this particular work well.

A comprehensive study of *the* two great factors in delinquency and crime—epilepsy and crime—would require many times over the space given to this discussion. As to epilepsy, I quote freely from the reports of Dr. William Healy, director of the Juvenile Psycopathic Institute of Chicago, who found 7.5 per cent of young repeaters in the courts definitely epileptic and others who were suspects but in whom no diagnosis had been made. The remarkable correlation between epilepsy and crime, he says, is due to two things:

(1) The disease itself usually produces a definitely characteristic mental and moral deterioration shown most markedly in the field of social inhibitory powers. This social inhibition being removed leaves him a victim to his own gross appetites, which result in viciousness and crime.

(2) The second is inherent in environmental conditions. Being socially and mentally inadequate, unwelcome and shunned in one's home, unable to hold a responsible job after a few seizures, even though his mental and moral deterioration has not become sufficiently advanced to contraindicate such trust, he is buffeted about, falls into bad associations, drifts with the current of the only social and moral world open to him, and eventually finds himself, as Dr. Healy says, "quite rudderless in a world which offers no chance for him." Taking the accepted estimate of 1:500, we should have in Dallas approximately 250 epileptics of either the major or minor types.

The advanced epileptic is especially unstable in the moral sphere, showing the greatest variations from day to day. Docile and obedient one day, he is a cunning or brutally vicious character the following, committing the most surprising and unexpected crimes of a widely varied character. Dr. Healy says that the gravest sex offenders coming under his observation and through the Chicago courts are epileptics. This is due, of course, to their premature and excessive sex development, plus their social inhibition and moral deterioration. Epileptic girls are particularly serious problems as far as sex delinquency is concerned. Knowing his limitations as we do, it is interesting to note that courts of appeal have decided that epilepsy is no excuse for crime unless the deed be done during a seizure. Early discovery of these cases, careful oversight, relief from mental strain, the prevention of procreation, adequate and specially adapted instruction and training, and eventually permanent custodial care in an institution designed especially for them is the only safe and humane disposition of this class of unfortunates. In its absence for these and the feebleminded as well, surgical sterilization may be, and probably is, of some real value, but to my mind it is only half a remedy and by no means a panacea for these social ills.

As to the big subject of feeble-mindedness, a prominent American says that "the trouble with this problem is that there are so many of us." We are partially exonerated, however, by the universally accepted definition of feeble-mindedness given by the royal commission of the Royal College of Physicians of London as "one who is capable of earning a living under favorable circumstances, but is incapable from mental defects existing from birth or from an early age (a) of competing on equal terms with his normal fellows, or (b) of managing himself or his affairs with ordinary prudence." While we may all be a little off, there is a lower limit beyond which the condition becomes significant. The census of 1890 obtained the ratio of 1:500 of our population as feebleminded by the enumerator simply asking the question, "Are there any idiots or imbeciles in your family?" Although a high percentage, one can easily see that from family pride, ignorance, etc., such an estimate must be far below the actual count. This has been more recently, and probably conservatively, estimated as 1:300, or even 1:250. Dr. Henry Goddard gives 1:242 for England; 1:187 for Michigan; 1:50 for an entire school system of 1000 children, and 1:32 for another of 800 examined by one of the authorities of Leland Stanford. He says that many have questioned this high percentage, but that no one has proved it incorrect, and that those who have begun to investigate have quickly discovered that it is not too high. Expressing doubt as to whether more careful and accurate investigations will place this percentage still higher, he says, "I believe that we are perfectly safe in considering that 1:200 of the population, or 2 per cent of the school children are so mentally defective as to be incapable of competing with their normal fellows or of managing themselves or their affairs with ordinary prudence." Upon an approximate enrollment of 25,000 children in the Dallas schools, we should have, if this estimate is correct and applies to us, about 500 feeble-minded children in our schools who need to be in special classes with thirty-five to fifty teachers in charge. In appealing for such specialized training Dr. Walter Fernald, director of the Massachusetts School for the Feeble-minded, says that "unless properly cared for and influenced they will retrograde, fall into evil ways, and become willing victims of the vicious. The boys and girls in our reform schools, the great army of police court chronic drunkards, and criminals, the tramps, vagrants, and low prostitutes, are largely recruited from this class of slightly mentally deficient who were neglected in their youths."

I believe from a purely financial and economic point of view that proper and adequate education and training for this portion of our citizenship is infinitely cheaper than the support of charity hospitals, alms-houses, poor farms, courts, and prisons for these and their numerous offspring—entirely aside from the dividends of human happiness paid on such an investment. As to institutional care, I have been able to find only three Southern States mentioned—Virginia, with an estimated feeble-minded population of 6872, cares for 74 in an institution; Kentucky has 7633, with 333 cared for, and Texas has 12,988, with none receiving institutional care. The last Legislature appropriated $50,000 per year for two years for the purpose of establishing a Farm Colony in the vicinity of Austin for the feeble-minded, drug fiends, and alcoholics. More than a year has passed and a board has not even been appointed nor an effort made to secure a location. Until recently it seems that the authorities have been too busy, if we are to take their word for it, to bother with this matter.

As to the causes of feeble-mindedness, they are numerous and need not be discussed here further than to emphasize that feeble-mindedness is due to an organic or structural brain defect, whether from heredity, disease in infancy or early life, or accident so, as Dr. Fernald says, "No really feeble-minded person ever was or ever can be cured." By training they may become "self-supporting," but not "self-controlling," and hence need oversight and supervision as long as they live. As to the connection between crime and feeble-mindedness, he considers every imbecile, especially the high-grade imbecile, a potential criminal—needing only the proper environment and opportunity for his criminal tendencies to develop. Malicious, mischievous children, runaways, vagrants, incorrigibles, the disorderly and ungovernable, are often of this imbecile type.

The Seattle juvenile court, whose reports merit our confidence inasmuch as it is one of the few that employs an expert psychologist, reports 18.5 per cent of their delinquencies due to feeble-mindedness, while Dr. Goddard says that not less than 25 per cent of the criminals that come before the courts are feeble-minded, that these criminals "are not born, they are made, and the material out of which they are made is feeble-mindedness." Fernald says that "at least 25 per cent of the inmates of our penal institutions are mentally defective and belong either to the feeble-minded or to the defective delinquent class"; that 50 per cent of the girls in the Lancaster Reform School, a great number of the neglected and dependent children of the State, a large percentage of prostitutes, of parents arraigned for the abuse of children, drunkards, and drug fiends, belong to the army of the feeble-minded. I am thoroughly convinced that a very large percentage of the girls in the various rescue homes are so feeble-minded as to be absolutely incapable of reform or of self-support. Early recognition of these cases would probably have kept them out of such homes, while recognition at the present time would no doubt convert much praying and many tears of pity into more active physical training of such faculties as they may possess. Of 105 mothers of illegitimate children examined in a Pennsylvania rescue home, 100 were found to be definitely feeble-minded.

The remedy for this social menace lies in part at least in an awakened conscience to the needs of this class of unfortunates, their early recognition by means of careful oversight in the homes where the parents themselves are intelligent and expert medical supervision in the schools for such other cases, probably the large majority, as would otherwise escape detection, adequate marriage laws, and other means to prevent procreation, the organization of special classes in our schools to give instruction suitable for their needs, instead of subjecting them to the torture of being ground out in the mill of every-day school life, surgical sterilization in some cases, and more sane and widespread teaching of the principles of eugenics as prophylactic measures, and adequate custodial care as a more immediate and alleviative measure.

We shall probably be more just and humanitarian in our attitude when we, as a public, come to think with Dr. Sylvester, that the criminal is not the desperate, vicious character the lay mind has pictured him, but rather the weakling whose powers of inhibition have been squandered by himself or his ancestors.

